# Correlation study on the risk of cardiovascular adverse events in diabetic foot patients based on machine learning - a retrospective cohort study

**DOI:** 10.3389/fendo.2025.1595471

**Published:** 2025-08-01

**Authors:** Liran Zheng, Jiageng Chen, Wenyan Xu, Min Ding, Juan Li, Fenghua Tian, Lei Zhang, Qianqian Li, Shuai Wang, Zeyu Wang, Hairong Ma, Xuecan Cui, Bai Chang, Meijun Wang

**Affiliations:** ^1^ NHC Key Lab of Hormones and Development and Tianjin Key Lab of Metabolic Diseases, Tianjin Medical University Chu Hsien-I Memorial Hospital & Institute of Endocrinology, Tianjin, China; ^2^ Human Resources Department, Tianjin Medical University, Tianjin, China

**Keywords:** diabetic foot, major adverse cardiovascular events, random forest mode, risk prediction, relevance

## Abstract

**Introduction:**

Diabetic Foot (DF), as a serious complication of diabetes, is closely related to major adverse cardiovascular events (MACE) and mortality. However, research on predictive models for the MACE risk in DF patients is not sufficient. The purpose of this study is to construct a prognostic model for the MACE risk in patients with diabetic foot ulcers and provide a reference tool for clinical individualized management.

**Method:**

This study retrospectively collected data of DF patients who were hospitalized and met the inclusion and exclusion criteria in a tertiary first-class comprehensive hospital mainly engaged in metabolic diseases in Tianjin from January 2018 to January 2020. The follow-up outcome was the occurrence of MACE within 5 years after discharge. Multiple imputation (MI) method was used to fill in the missing data. Based on the processed data, in terms of modeling methods, the top three frequently used methods were used. Logistic regression, random forest (RF) and support vector machine (SVM) were used respectively to analyze influencing factors. The performance of each model was compared by using confusion matrix, ROC curve and AUC value. The data set was divided into training set and test set according to the proportion of 80%/20%. Finally, the model effect was verified on the test set. The study finally included a total of 504 patients with DF. Among them, 147 cases (29.17%) experienced MACE events within five years. The AUC of the RF model in this study was 0.70, the AUC of the Logistic regression model was 0.62, and the AUC of the SVM model was 0.60.

**Conclusion:**

All three models established in this research have good clinical predictive ability. Among them, the clinical prediction model based on RF has the best effect and can effectively predict the risk of MACE in DF patients, helping clinical medical staff formulate personalized treatment plans.

## 1 Introduction

DF is one of the serious complications of diabetes. It is based on diabetes and causes abnormal lower extremity nerves and/or distal peripheral vascular lesions in patients, leading to foot infections, ulcers and/or destruction of deep tissues. It has the characteristics of difficult treatment, poor prognosis, high disability and mortality rates (1). Compared with diabetic patients without foot complications, the death risk of diabetic patients with DF is increased by 2.5 times, and the 5-year mortality rate is 42% (2). Among them, MACE is the main cause of morbidity and death in patients with a history of diabetic-related foot ulcers (3–5). The average age of patients who have had DF is 5 years lower than that of DF patients when cardiovascular events occur (5). Therefore, DF is not only representative of severe complications of diabetes, but is also recognized as an important indicator of the incidence and mortality of MACE. Previous literature has shown that DF patients have more than twice the risk of MACE and death than non-DF patients (6). Although the risk association between DF and MACE has received certain attention, the research on predictive models for MACE risk in DF patients is still relatively limited at present. Traditional prediction models are mostly based on Cox regression or Logistic regression. These methods have limitations in dealing with nonlinear relationships and complex interaction effects between variables (7). At the same time, existing cardiovascular risk prediction tools, such as the Framingham risk score (8) and the UKPDS risk engine (9), mainly target general diabetic populations and have not been specially adjusted for DF patients. Their prediction effects may not be able to fully reflect the characteristics of this high-risk group of DF. In recent years, machine learning methods have gradually been applied to medical risk prediction research due to their advantages in processing high-dimensional data and capturing nonlinear relationships (10). For this purpose, this study comprehensively considered three models: Logistic regression, Support Vector Machine (SVM), and Random Forest (RF). Based on previous studies (11–13), it integrated data from multiple aspects such as demographic characteristics, duration of diabetes, blood biochemical indicators, and complications to optimally construct a model for predicting the risk of MACE after discharge of DF patients. We used indicators such as confusion matrix, ROC curve, and AUC value to evaluate the prediction ability of the models constructed by the three methods. The aim is to develop a personalized prognostic scoring system that can automatically identify nonlinear relationships and interactions between variables and has good stability and clinical interpretability, so as to provide data support and decision-making basis for the risk stratification and individualized treatment of clinical DF patients.

### 1.1 Research design and methods

This study retrospectively included DF patients who met the inclusion and exclusion criteria and were hospitalized in a tertiary first-class comprehensive hospital mainly focusing on metabolic diseases from January 2018 to January 2020. The follow-up of all patients ended on December 30, 2024.

**Figure 1:**
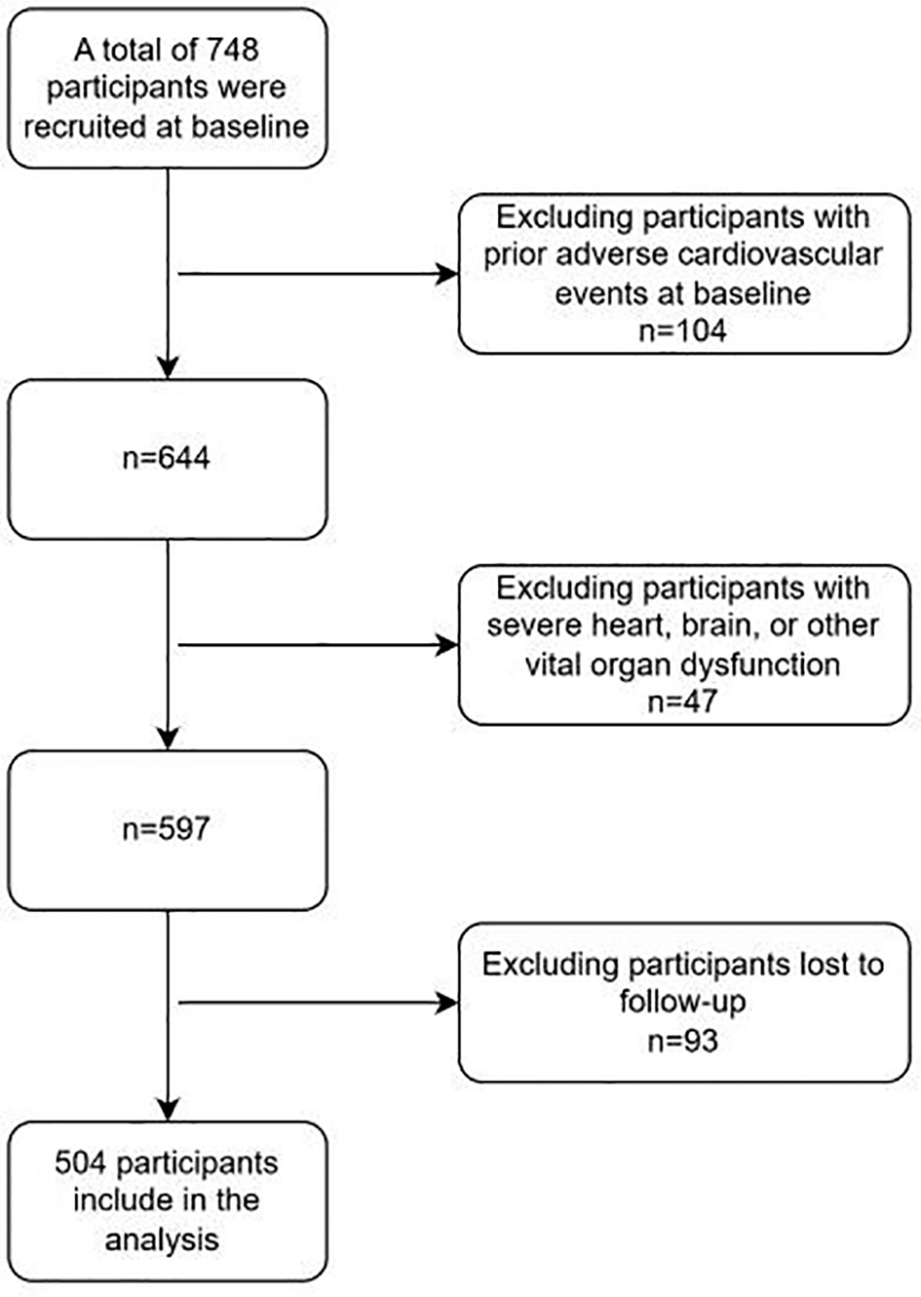
Inclusion and exclusion criteria.

Inclusion criteria: (1) Diabetic patients meet the relevant diagnostic criteria in “Chinese Guidelines for the Diagnosis and Treatment of Elderly Diabetes (2019 Edition)” (14),namely: typical diabetes symptoms (excessive thirst, frequent urination, increased appetite, unexplained weight loss) plus random venous plasma glucose >11.1 mmol/L; or plus fasting venous plasma glucose >7.0 mmol/L; or plus venous plasma glucose >mmol/L two hours after glucose loading. For those without typical diabetes symptoms, should be re-examined on another day for confirmation. The WHO recommends using a hemoglobin A1c level >6.5% as the diagnostic cutoff for diabetes in countries and regions where conditions permit.(2) The diagnostic criteria for Wagner grade of DF meet “Chinese Guidelines for the Prevention and Treatment of Diabetic Foot (2019 Edition) (II)”, and the patients’ Wagner grades are all above grade 2 (15). Specifically, as follows, Grade 0: At risk foot, but no ulceration present; Grade 1: Superficial ulcer involving the full thickness of the epidermis but not underlying tissues; Grade 2: Deeper ulcer extending into ligaments and muscle, but no involvement of bone or abscess formation; Grade 3: Deep infection with involvement of bone (osteomyelitis) or abscess formation; Grade 4: Localized gangrene (e.g., toes, forefoot); Grade 5: Extensive gangrene involving the whole foot. (3) Patients who received treatment for DF in our hospital from January 2018 to January 2021. (4) The diagnosis of recurrent DF ulcer mainly refers to the 2019 guidelines formulated by the International Working Group on DF Ulcers (IWGDF) (16). Exclusion criteria: (1) Having experienced adverse cardiovascular events; (2) Failed to follow up and unable to cooperate with the completion of follow-up; (3) Combined with serious dysfunction of important organs such as the heart and brain. A total of 748 patients with DF were included in this study. After inclusion and exclusion, a total of 504 patients entered the research analysis. (See **Figure 1**).

In order to fully evaluate the risk predictors of cardiovascular adverse events in DF patients, this study included risk factors from categories such as general information, biochemical indicators, medical history, and other comorbidities. These included general information such as age, gender, body mass index (BMI), etc., systolic blood pressure (SBP), diastolic blood pressure (DBP), fasting blood glucose, 2-hour postprandial blood glucose, glycosylated hemoglobin, etc., blood tests such as coagulation function, total blood lipid, liver and kidney function, as well as previous history of foot ulcers, surgical history, and other comorbidities of diabetes. Obtained through methods such as reviewing medical records, readmission records, and telephone follow-ups, including information on cardiovascular events, death, survival, refusal to be interviewed, and loss to follow-up. This study has been approved by the hospital ethics committee and strictly follows the Helsinki Declaration and relevant ethical standards. Given that this study is a retrospective analysis, the ethics committee has waived the requirement for patients to sign informed consent forms.

### 1.2 Follow-up

In this study, 504 patients diagnosed with DF were followed up regularly. The end point of follow-up was the first occurrence of MACE within 5 years. Cardiovascular events included cardiogenic death, myocardial infarction, angina pectoris attack, heart failure, redo revascularization, malignant arrhythmia, stent thrombosis, etc. (17), with the doctor’s diagnosis result as the standard. The disease diagnosis was defined by the International Classification of Diseases, Tenth Revision (ICD-10). Before retrospectively collecting relevant information of patients, the follow-up personnel were trained, and the information was checked on the day when data entry ended. When collecting follow-up information, the outcome information and time were recorded, and the information was collected through outpatient medical records, re-admission records and telephone follow-up.

### 1.3 Statistical methods

Statistical analysis was performed using SPSS 26.0 and R 4.2.1. Measurement data were expressed using quartiles [Md (P25, P75)]. Count data were described using frequency and composition ratio. The difference comparison of measurement data was analyzed using t-test or U-test, and the count data was analyzed using χ2-test. The test level α = 0.05. The patient outcome was assigned a value (MACE). The occurrence of MACE events was set as 1, and the non-occurrence was set as 0. Through detection, it was found that most variables had missing values. The number of missing values of transcutaneous oxygen partial pressure left and transcutaneous oxygen partial pressure right was too large, close to 50%, and it was not suitable for data filling. Therefore, the variables were deleted. The bare ankle index (left) and the bare ankle index (right) have a large number of missing values, which are 66.7% and 67.9% respectively. However, the missing rate does not exceed 40%.The missing rates of other variables do not exceed 10%.Therefore, the multiple imputation method (18) (Multiple Imputation, MI) is selected to fill in the missing data.

Multiple imputation (MI) is an important statistical method for dealing with missing data. It is based on the distribution characteristics of data and fills in missing values multiple times by establishing models to generate multiple complete data sets. Each data set considers the randomness of data when filling in. Then, the analysis results of multiple data sets are integrated to obtain more stable and reliable statistical estimates. Compared with single imputation methods, it can better reflect the uncertainty of data, reduce the bias caused by missing data. It is widely used in social science research, medical data processing and other fields, and effectively improves the accuracy and scientific nature of data analysis.

The purpose of this study is to analyze the influencing factors of outcomes. Logistic regression (19), Random Forest (20) (Random Forest, RF), and Support Vector Machine (21) (Support Vector Machine, SVM) were selected for analysis. In order to select the optimal factor judgment method, this study intends to use three indicators, namely Confusion Matrix (CM), ROC curve, and AUC, to compare the three methods and use the optimal method to judge the influencing factors. In order to ensure the effect of the final model, this study splits the data. 80% of the data is used as training data and 20% as test data. The final output result is the result of the test data running in the trained model.

## 2 Result

There is no difference in the distribution of data before and after multiple interpolation. See **Supplementary Figure 1** for a diagram of missing value patterns. A comparison of baseline data between DF patients and cardiovascular adverse events.

This study included a total of 504 patients. Among them, there were 147 cases (29.17%) in the MACE group and 357 cases (70.83%) in the non-MACE group. There were statistically significant differences between the two groups in multiple baseline characteristics, including age (P = 0.001), diastolic blood pressure (P = 0.043), D-dimer (P = 0.022), urinary microalbumin (P < 0.001), total urinary protein (P < 0.001), serum creatinine (P = 0.003), gender (P = 0.047), aspirin usage (P < 0.001), and the combined situation of diabetic peripheral neuropathy (P = 0.027). In addition, there were also significant differences in red blood cell count, C-reactive protein and left ankle-brachial index between the two groups, suggesting that these factors may be related to the occurrence of MACE. See **Table 1** for details.

**Table 1:** Comparison of baseline data characteristics between the MACE group and the non-MACE group.

Variable	Total (n = 504)	MACE (n = 147)	Nonoccurance MACE (n = 357)	*P*值
Age	63.08 ± 12.08	65.61 ± 10.89	62.04 ± 12.40	**0.001**
BMI	25.31 ± 4.09	25.41 ± 3.99	25.27 ± 4.14	0.726
BUN	6.73 ± 3.90	7.04 ± 3.48	6.60 ± 4.06	0.253
Fibrinogen	3.97 ± 1.56	4.14 ± 1.49	3.89 ± 1.58	0.100
HbA1c	8.99 ± 2.09	8.90 ± 2.07	9.03 ± 2.10	0.524
SBP	138.49 ± 19.96	140.88 ± 22.15	137.51 ± 18.93	0.108
DBP	77.55 ± 10.02	76.14 ± 10.55	78.13 ± 9.75	**0.043**
FBG	8.78 ± 3.47	8.59 ± 3.51	8.86 ± 3.46	0.425
2hBG	12.76 ± 4.00	12.66 ± 4.04	12.80 ± 3.99	0.730
TG	1.47 ± 0.79	1.50 ± 0.90	1.46 ± 0.74	0.548
TC	4.38 ± 1.23	4.42 ± 1.21	4.36 ± 1.24	0.654
LDL	3.02 ± 1.00	3.12 ± 0.98	2.97 ± 1.00	0.124
HDL	0.99 ± 0.33	1.00 ± 0.28	0.98 ± 0.36	0.704
Diabetes duration	15.03 ± 8.74	15.92 ± 8.47	14.66 ± 8.83	0.142
D-dimer	0.58 (0.36, 1.02)	0.72 (0.39, 1.21)	0.56 (0.34, 0.89)	**0.022**
24h UMI	91.80 (18.19, 300.00)	259.92 (29.13, 420.52)	71.12 (16.10, 300.00)	**<.001**
24h UTP	0.34 (0.10, 1.76)	0.80 (0.12, 2.70)	0.21 (0.10, 1.43)	**<.001**
SCR	71.25 (58.50, 90.92)	77.30 (62.85, 97.95)	67.80 (57.70, 88.40)	**0.003**
Recurrence				0.106
NO	422 (83.73)	117 (79.59)	305 (85.43)	
YES	82 (16.27)	30 (20.41)	52 (14.57)	
Ulcerated area				0.195
Acrotarsium	411 (81.55)	125 (85.03)	286 (80.11)	
Pelma	93 (18.45)	22 (14.97)	71 (19.89)	
Sex				**0.047**
Female	150 (29.76)	53 (36.05)	97 (27.17)	
Male	354 (70.24)	94 (63.95)	260 (72.83)	
Taking aspirin drugs				**<.001**
NO	310 (61.51)	74 (50.34)	236 (66.11)	
YES	194 (38.49)	73 (49.66)	121 (33.89)	
Taking statin drugs				0.322
NO	288 (57.14)	79 (53.74)	209 (58.54)	
YES	216 (42.86)	68 (46.26)	148 (41.46)	
Smoking				0.902
NO	255 (50.60)	75 (51.02)	180 (50.42)	
YES	249 (49.40)	72 (48.98)	177 (49.58)	
History of previous amputation				0.252
NO	236 (46.83)	63 (42.86)	173 (48.46)	
YES	268 (53.17)	84 (57.14)	184 (51.54)	
Previous foot ulcer				0.724
NO	275 (54.56)	82 (55.78)	193 (54.06)	
YES	229 (45.44)	65 (44.22)	164 (45.94)	
Foot deformity				0.133
NO	434 (86.11)	121 (82.31)	313 (87.68)	
Flat foot, and bunions	53 (10.52)	17 (11.56)	36 (10.08)	
Hallux valgus	7 (1.39)	4 (2.72)	3 (0.84)	
3	10 (1.98)	5 (3.40)	5 (1.40)	
Retinopathy				0.837
NO	223 (44.25)	64 (43.54)	159 (44.54)	
YES	281 (55.75)	83 (56.46)	198 (55.46)	
DPN				**0.027**
NO	11 (2.18)	7 (4.76)	4 (1.12)	
YES	493 (97.82)	140 (95.24)	353 (98.88)	
Diabetes treatment				0.572
Oral medicine	184 (36.51)	49 (33.33)	135 (37.82)	
Insulin	70 (13.89)	23 (15.65)	47 (13.17)	
Oral combination with insulin	250 (49.60)	75 (51.02)	175 (49.02)	
RBC, Mean ± SD	4.02 ± 0.72	4.08 ± 0.70	3.86 ± 0.77	<0.001
WBC, M (Q_1_, Q_3_)	7.19 (5.57, 9.02)	7.13 (5.56, 8.73)	7.42 (5.58, 9.70)	0.42
PLT, M (Q_1_, Q_3_)	251.50 (191.25, 335.00)	246.00 (187.50, 331.00)	257.00 (201.00, 336.50)	0.320
CRP, M (Q_1_, Q_3_)	9.70 (2.10, 19.70)	7.10 (1.65, 17.80)	12.55 (3.30, 24.84)	0.04
Foot temperature left, M (Q_1_, Q_3_)	34.80 (33.70, 35.50)	34.80 (33.70, 35.40)	34.80 (33.80, 35.60)	0.842
Foot temperature right, M (Q_1_, Q_3_)	34.60 (33.68, 35.40)	34.60 (33.80, 35.40)	34.30 (33.50, 35.40)	0.272
ABI left, M (Q_1_, Q_3_)	1.03 (0.79, 1.16)	1.06 (0.85, 1.17)	0.93 (0.69, 1.13)	0.006
ABI right, M (Q_1_, Q_3_)	1.02 (0.80, 1.15)	1.04 (0.81, 1.15)	0.96 (0.75, 1.13)	0.101
Vibration, n(%)				
1	503 (99.80)	356 (70.78)	147 (29.22)	
MISS	1 (0.20)	1 (100.00)	0 (0.00)	
Proprioception, n(%)				
1	503 (99.80)	356 (70.78)	147 (29.22)	
MISS	1 (0.20)	1 (100.00)	0 (0.00)	
Thermoception, n(%)				0.893
1	502 (99.60)	356 (70.92)	146 (29.08)	
MISS	2 (0.40)	1 (50.00)	1 (50.00)	
Pain, n(%)				
0	2 (0.40)	2 (100.00)	0 (0.00)	
1	501 (99.40)	354 (70.66)	147 (29.34)	
MISS	1 (0.20)	1 (100.00)	0 (0.00)	
Dorsalispedisartery, n(%)				
1	503 (99.80)	356 (70.78)	147 (29.22)	
MISS	1 (0.20)	1 (100.00)	0 (0.00)	
Osteomyelitis, n(%)				0.425
0	288 (57.14)	200 (69.44)	88 (30.56)	
1	211 (41.87)	154 (72.99)	57 (27.01)	
MISS	5 (0.99)	3 (60.00)	2 (40.00)	

Bold values mean P <0.05 was statistically significant.

We conducted a feature correlation analysis of the model variables and found no VIF between the variables (**Supplementary Figure 2**). In this study, we used confusion matrices to further compare the performance of three classification models in predicting the occurrence of major adverse cardiovascular events (MACE) in DF patients (**Tables 2**–**4**).

**Table 2:** Logistic regression confusion matrix.

True value	Prediction result		In total
	0	1	
0	63	6	69
1	23	9	32
In total	86	15	101

**Table 3:** RF confusion matrix.

True value	Prediction result		In total
	0	1	
0	63	6	69
1	25	7	32
In total	88	13	101

**Table 4:** SVM confusion matrix.

True value	Prediction result		In total
	0	1	
0	68	1	69
1	31	1	32
In total	99	2	101

The confusion matrix of Logistic regression is shown in **Table 2**.The prediction accuracy rate is 71.2%.

The confusion matrix of Random Forest (RF) is shown in **Table 3**. The prediction accuracy rate is 69.3%.

The confusion matrix of Support Vector Machine (SVM) is shown in **Table 4**. The prediction accuracy rate is 68.3%.

**Figure 2:**
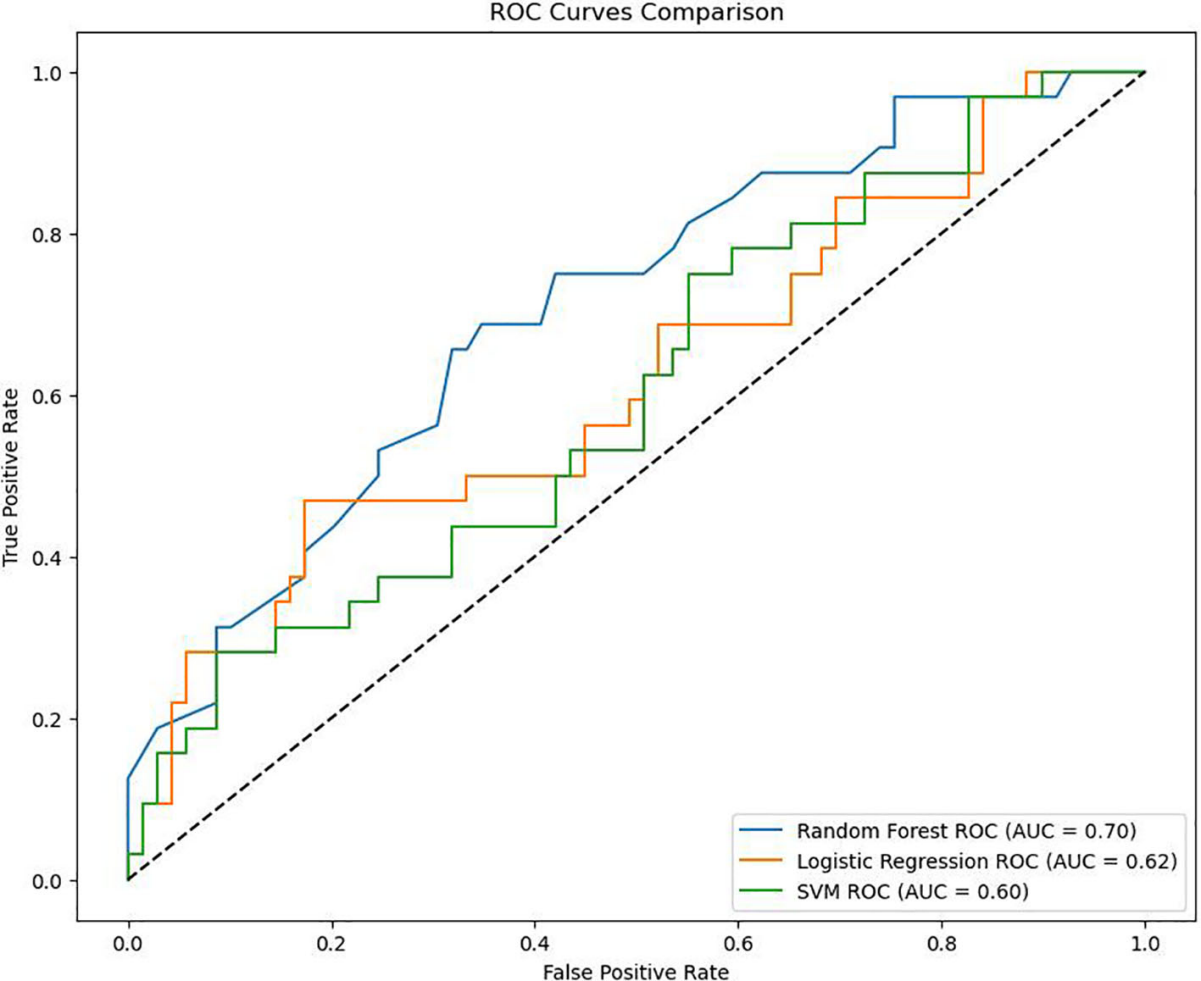
ROC curve graph.

**Figure 3:**
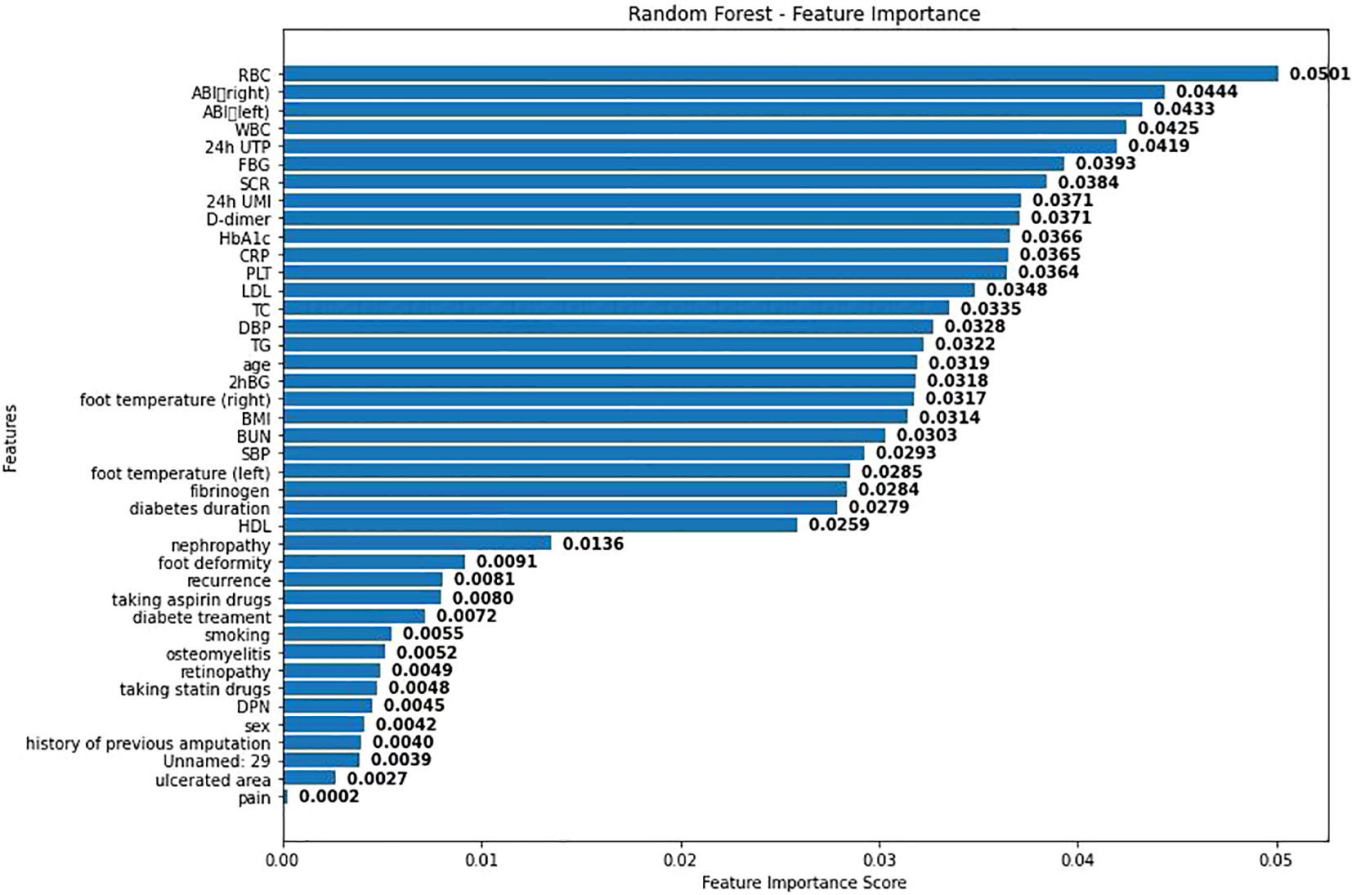
RF variable importance diagram.

The ROC curves drawn by the three models are shown in **Figure 2**. As shown in **Figure 2**, the AUC of RF in this study is 0.70, the AUC of Logistic regression is 0.62, and the AUC of SVM is 0.60. Considering that the data in this study are based on real-world clinical cases, it can be considered that the three models established in this study all have good clinical prediction ability. And the clinical prediction model based on RF has the best effect. Therefore, this study further ranks the importance of each variable in RF as shown in **Figure 3**.

As shown in **Figure 3**, visualization was performed according to the importance degree of variables during the establishment process of RF, and the feature importance scores obtained from the random forest model were displayed. Each bar represents a different feature, and the length of the bar indicates the relative importance of this feature in predicting adverse cardiovascular events. Among them, the red blood cell count has the highest importance score, indicating that it has the greatest impact on the model prediction results. This may be because anemia will lead to poor prognosis of DF patients. Secondly, BMI and age also show high influence.

## 3 Discussion

This study reveals that the risk of MACE in DF patients is significantly higher through three methods: Logistic regression, random forest, and support vector machine. This finding indicates that DF is not only a manifestation of severe complications but also an important sign of the risk of adverse cardiovascular events. Secondly, the study points out that among the three statistical methods adopted, the random forest model performs best. This may benefit from the advantages of random forest in dealing with nonlinear relationships and complex interactions, so that this model is more accurate in predicting the risk of adverse cardiovascular events in DF patients.

The high incidence rate of cardiovascular adverse events observed in DF patients in this study is consistent with the results of previous studies. The potential mechanisms of this association are multifactorial: Firstly, the onset of DF is closely related to microvascular diseases (such as peripheral neuropathy) and macrovascular diseases (such as PAD) (22). Secondly, previous systematic reviews and meta-analyses have shown that the risk of cardiovascular death in patients with DF is more than twice higher than that in non-DF patients (23, 24). This increased risk may be related to the following mechanisms: (1) The inflammatory response caused by persistent infection promotes endothelial dysfunction, oxidative stress and atherosclerosis; (2) The increased risk of thrombosis leads to an increased risk of myocardial infarction or stroke (25); (3) The chronic inflammatory state can induce myocardial fibrosis and cardiac vascular remodeling, ultimately leading to heart failure (26).

In addition, this study found that there were significant differences in several clinical characteristics between the MACE group and the non-MACE group. For example, the age difference was consistent with previous studies. Among them, a large cohort study by Huang et al. (27) (n = 8,514) showed that for every 10-year increase, the risk of cardiovascular events in DF patients increased by 1.4 times. This may be related to the aggravation of vascular lesions and the decline of organ function reserve in older patients. In terms of cardiovascular-related indicators, the lower diastolic blood pressure and left ankle-brachial index in the MACE group reflected the potential vascular function damage of patients. A systematic review by Brownrigg et al. (28) found that the risk of cardiovascular events in DF patients with ABI < 0.9 was 2.7 times that of patients with normal ABI. This study also observed that multiple clinical biochemical indicators in the MACE group had a significant impact on the risk of its occurrence, such as D-dimer, C-reactive protein, microalbuminuria, total urinary protein and serum creatinine, etc. This is consistent with the impact of chronic inflammation and abnormal coagulation function on cardiovascular diseases reported in previous studies (29).

It is worth noting that the findings of this study lie in that the differences in red blood cell count also have statistical significance. Although there is currently a lack of direct research evidence, the study by Sun et al. (30) suggests that anemia may be one of the risk factors for poor prognosis in patients with DF.

In addition, all patients in this study generally had symptoms such as weakened vibration sensation, proprioception, dorsalis pedis artery pulsation and temperature sensation. This is consistent with the disease progression described by Paisey et (31). This study also emphasizes the importance of early neuropathy screening in preventing cardiovascular complications.

Overall, the comparison results of the baseline characteristics in this study are basically consistent with those reported in previous literature. At the same time, a new potential risk factor, namely red blood cell count, was discovered. This finding provides an important basis for further improving the risk prediction model and optimizing prevention and treatment strategies. In terms of statistics, this research innovatively applies three machine learning methods. Logistic regression, Random Forest (RF), and Support Vector Machine (SVM) are selected for analysis. At the same time, in order to select the optimal factor judgment method, three indicators, Confusion Matrix (CM), ROC curve, and AUC, are also used to compare the three methods. And the optimal method is used to judge the influencing factors. The model can be used to construct a clinical prognosis scoring system, providing doctors with an objective and quantified risk assessment tool to optimize clinical decision-making. At the same time, based on the individualized cumulative risk curve of the model, it helps patients understand their own risks and enhance the initiative and treatment compliance of disease management. In addition, this model can be used as a reference for formulating personalized follow-up strategies and preventive intervention measures to help clinical medical staff accurately identify high-risk populations, thereby optimizing long-term management plans and reducing the risk of adverse events.

In addition, this study still has certain limitations. Firstly, this study is a single-center DF study and requires further multi-center clinical cohorts to improve the represent activeness of samples and thus test the generalizability of the model and scoring. Secondly, the clinical variables included in this study are mainly complication and biochemical examination information of patients. In the follow-up study, it is planned to increase the collection and analysis of postoperative indicators of DF patients, and make good statistical years and risk stratification for the occurrence of events to improve the stability of the model and the reliability of the scoring. The study was a cross-sectional study and could not determine causality or sequence before and after, In the future, multi-center prospective cohort studies can be carried out to expand the sample size and improve the representativeness. At the same time, more clinical relevant factors, especially surgical related indicators and dynamic variables during the follow-up process, can be included. A mobile medical platform based on this model can also be established to achieve the automation of risk prediction. Lastly, When conducting the MCAR test, the covariance matrix was singular, possibly because the missing data pattern did not meet the MCAR assumption conditions, or because the data set was too small or the correlations between variables were too complex, making it impossible to obtain meaningful test results. So in the future we should further expand the sample size or conduct external validation to ensure the reliability of the data.

In conclusion, this study clarifies the potential mechanisms of high-risk MACE in DF patients and establishes an RF model with good predictive performance. This model integrates multiple clinical risk factors and can provide decision support for the precise management of DF patients. In the future, it is necessary to further improve and verify this predictive tool and explore its best application mode in clinical practice.

## Data Availability

The original contributions presented in the study are included in the article/**Supplementary Material**. Further inquiries can be directed to the corresponding authors.
