# Supplementary material for: Correlation study on the risk of cardiovascular adverse events in diabetic foot patients based on machine learning - a retrospective cohort study
Source: Front Endocrinol (Lausanne). 2025 Aug 1;16:1595471. doi: 10.3389/fendo.2025.1595471 (PMC12353740; doi:10.3389/fendo.2025.1595471)
Supplement: Supplementary file 1 [file DataSheet1.pdf]

A：

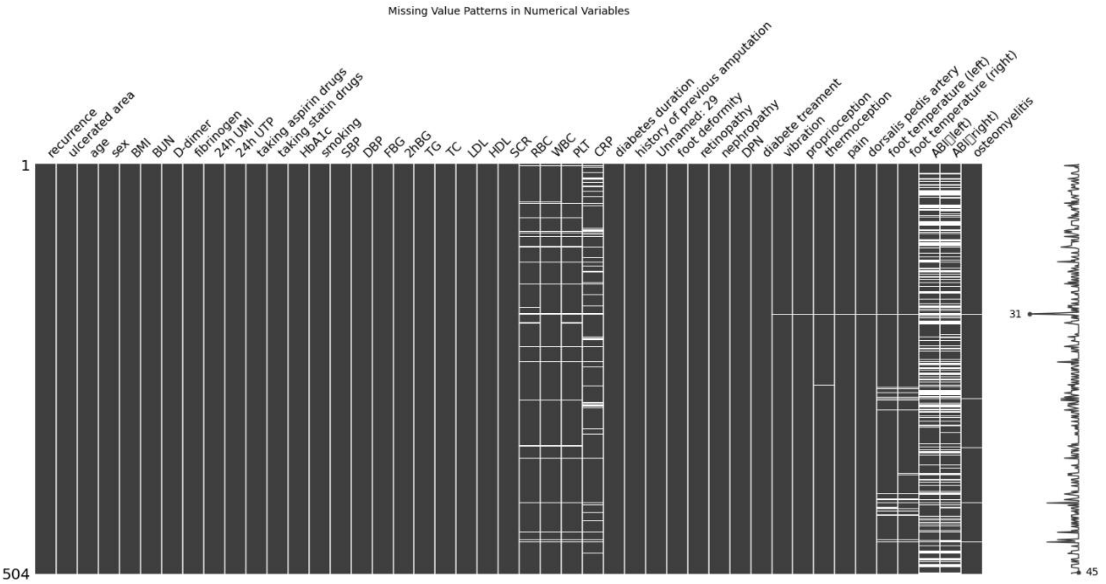

B：

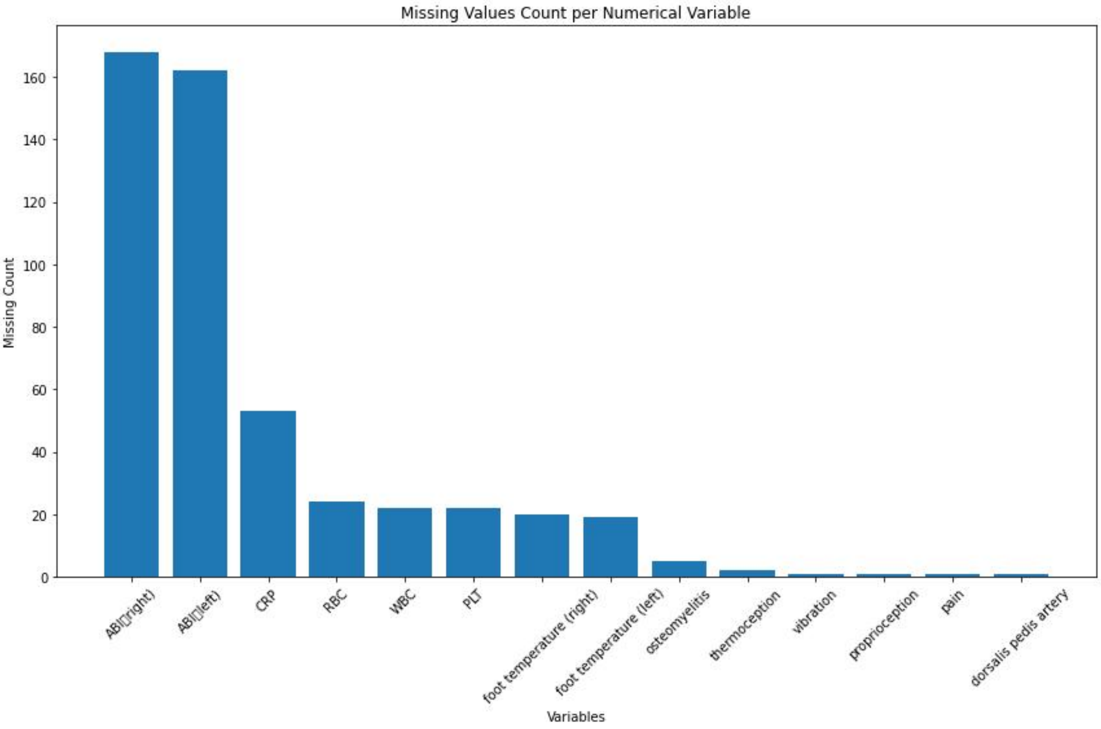

Supplyment Fig.1 Missing value patterns diagram.

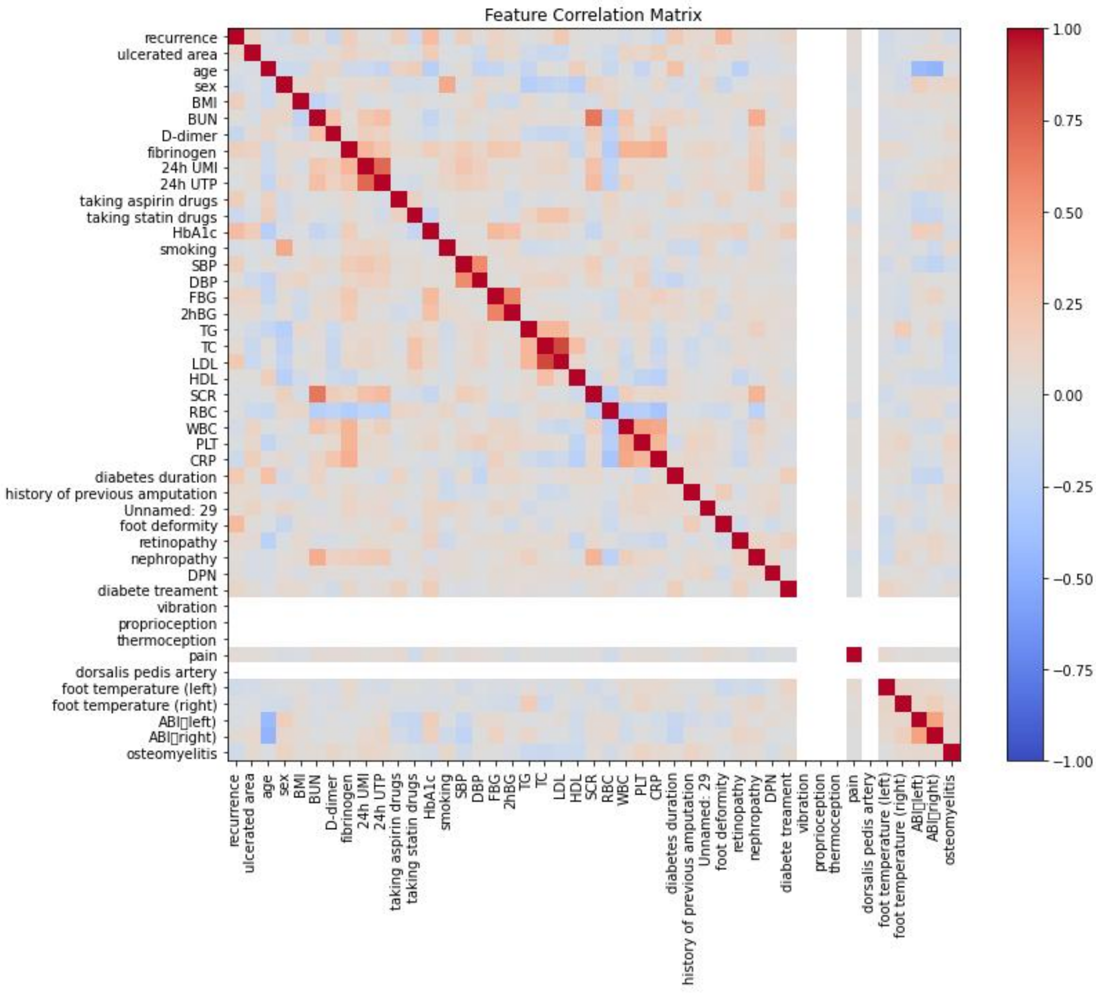

Supplyment Fig.2 Results of feature correlation analysis of model variables.
